# Protein localization of aquaporins in the adult female disease vector mosquito, *Aedes aegypti*

**DOI:** 10.3389/finsc.2024.1365651

**Published:** 2024-04-18

**Authors:** Britney Picinic, Jean-Paul V. Paluzzi, Andrew Donini

**Affiliations:** Department of Biology, York University, Toronto, ON, Canada

**Keywords:** water, osmoregulation, transport, protein expression, blood feeding

## Abstract

The female *Aedes aegypti* mosquito is a vector for several arboviral diseases, due to their blood feeding behavior and their association with urban communities. While ion transport in *Ae. aegypti* has been studied, much less is known about mechanisms of water transport. Rapid water and ion excretion occurs in the adult female mosquito post blood meal and involves a set of organs including the midgut, Malpighian tubules (MTs), and hindgut. The MTs are responsible for the formation of primary urine and are considered the most important site for active transport of ions. Within the cells of the MTs, along with various ion transporters, there are aquaporin water channels that aid in the transport of water across the tubule cell membrane. Six aquaporin genes have been molecularly identified in *Ae. aegypti* (AQP1–6) and found to be responsible for the transport of water and in some cases, small solutes such as glycerol. In this study, we used immunohistochemistry to localize AaAQP1, 2, 4, 5, and 6 in the adult female *Ae. aegypti*, in non-blood fed and post blood feeding (0.5 and 24hr) conditions. We further examined the main water transporting aquaporin, AaAQP1, using western blotting to determine protein abundance changes in isolated MTs pre- and post-blood feeding. Using fluorescence *in situ* hybridization, *aqp1* mRNA was found exclusively in the principal cells of female MTs. Finally, we used immunogold staining with transmission electron microscopy to determine subcellular localization of AaAQP1 in the Malpighian tubules under non-blood fed conditions. Interestingly, AaAQP1 was found to be predominantly in the principal cells of the MTs, dispersed throughout the brush border; however, there was also evidence of some AaAQP1 localization in the stellate cells of the MTs.

## Introduction

The mosquito, *Aedes aegypti* is responsible for the spread of deadly arboviral diseases such as Zika virus (1), Chikungunya (2), yellow fever (3), and dengue fever (4). Adult *Ae. aegypti* are terrestrial and feed on plant nectar for essential nutrients. Females also obtain a blood meal from vertebrate hosts to utilize the protein for egg maturation (5). Upon the initiation of blood feeding, female mosquitoes intake a large quantity of water and ions, which must be dealt with quickly. Rapid excretion of water and ions, such as Na^+^ and K^+^, has been shown to begin before a female *Ae. aegypti* has finished taking a blood meal (6, 7). Insects, including *Ae. aegypti*, have specialized organs that are responsible for osmoregulation and rapid urine excretion, namely the Malpighian tubules (MTs) and the hindgut. The MTs are comprised of two main epithelial cell types; the more abundant principal cells and the intercalated stellate cells that together are responsible for the production of primary urine, which is driven by an apical V-type H^+^-ATPase (VA) (8). Specifically, the VA maintains a proton gradient that is needed for secondary active transport of ions such as Na^+^ and K^+^ into the tubule lumen, in exchange for protons through cation proton antiporters expressed on the apical membrane of both cell types (9–11). In addition to the MTs, the posterior region of the hindgut (ie. rectum) is responsible for reabsorption of water and ions before final waste excretion (12). The excretion of ions has been studied extensively, however there is much less known about the movement of water by insect excretory organs, including the MTs.

Aquaporins are transmembrane proteins that form selective channels for water and some solutes such as glycerol and trehalose (13), that make it possible for the transcellular flow of water to occur. In *A. aegypti*, there have been six aquaporin genes identified [AaAQP1–6] (13–15). In this study, the AQP nomenclature used follows that of the Hansen lab (**Table 1**) (16). Functional characterization in a heterologous system revealed AaAQP 1, 2, and 5 allow significant water permeability (13) as does AaAQP6 (17), while AaAQP 4 and 5 have been identified as entomoglyceroporins, able to transport some solutes across the cell membrane (13). Sequencing (14), heterologous expression data (13), and gene replacement data (18) suggest that AaAQP4 and AaAQP5 have a different amino acid composition resulting in a larger pore diameter, allowing the transport of solutes like glycerol, urea, erythritol, adonitol, mannitol, and trehalose in addition to water. AaAQP5 has a comparatively high permeability to water, similar to AaAQP1, which is significantly higher than AaAQP4 that is a poor water transporter (13).

**Table 1:** Sizes and functional characteristics of aquaporins examined in the current study found in *Aedes aegypti* along with their orthologs in the African malaria vector mosquito, *Anopheles gambiae*, and the fruit fly, *Drosophila melanogaster*.

Name in *Aedes aegypti*	Accession Number	Length	Putative AQP Function	*Anopheles gambiae* Homolog	*Drosophila melanogaster* Homolog
AaAQP1	XP_001656931	249	Water-selective AQP	XP_319584	DRIP – CG9023
AaAQP2	XP_001649747	264	Water-selective AQP	XP_319585	PRIP – CG7777
AaAQP4	XP_001650168	292	Entomoglyceroporin	XP_554502	Eglp2 – CG5398
AaAQP5	XP_001650169	249	Entomoglyceroporin	XP_318238	Eglp4 – CG4019
AaAQP6	XP_001648046	261	Water-selective AQP	XP_309823	CG12251

Table data, including accession numbers, was compiled based on phylogenetic data described previously (16).

AaAQP1, 2, 4, 5, and 6 mRNA has been identified in the MTs of non-blood fed females (16). A blood meal in female *Ae. aegypti* increases mRNA levels of AaAQP1, 4, and 5 in the MTs, specifically between 3–48hr post-blood meal (13, 16). Previous work done in larval *Ae. aegypti* established the localization of AaAQPs throughout the alimentary canal with abundance of AaAQP1, 4, and 5 shown in the MTs (19), as well as in adult *Ae. aegypti* where AaAQP1 was localized to the tracheolar cells (20). To date, there has been no comprehensive characterization of AQPs in the adult *Ae. aegypti* mosquito; particularly at the protein level. In addition, identifying mechanisms by which AQPs in *Ae. aegypti* are regulated is important for understanding how a blood meal in female *Ae. aegypti* may affect localization, abundance and ultimately the function of AaAQPs. In *Ae. aegypti*, control and modification of MT function involves circulating hormones, including neuropeptides (21). The rate of fluid secretion by MTs increases with application of the neuropeptide, diuretic hormone 31 (DH_31_) and decreases with application of the anti-diuretic hormone, CAPA (21). The actions of DH_31_ and CAPA are mediated by intracellular signaling that involves assembly and disassembly of the VA in the apical membrane of principal cells (22). Through the control of fluid secretion by the MTs, AaAQP regulation is also possible, however there have been limited studies on invertebrate AQP regulation. It has been proposed that AQPs can be phosphorylated during periods of stress, in addition to the possibility that they are packaged into membrane vesicles on demand, for example during diuresis (23). The goal of this study was to provide a deeper understanding of the localization of AQPs in the adult female *Ae. aegypti* and to gain better insight on AaAQP1 expression and regulation before and after blood feeding by female mosquitoes.

## Materials and methods

### Mosquito rearing

*Aedes aegypti* eggs (Liverpool) were gathered from a long-standing colony reared at York University in Toronto, Ontario Canada. Filter paper was placed in small cups filled with ddH_2_O, where adult females were able to lay their eggs at the surface of the water. Females were fed twice weekly with sheep’s blood in Alsever’s solution (Cedarlane Laboratories, Burlington, Ontario Canada) using an artificial feeding method (24). Egg strips were then air dried and hatched as necessary, in 1L dechlorinated water baths. Larvae were fed with a 1:1 liver powder-yeast mixture dissolved in ddH_2_O. As the larvae pupated, the pupae were collected in small 10mL containers and placed in mosquito cages. Each mosquito cage was provided with a 10% sucrose-soaked cotton ball to allow the mosquitoes to feed on a simulated nectar meal *ad libitum*. Male and female pupae were combined in each mosquito cage. For non-blood fed conditions, ~10–15 female *Ae. aegypti* were isolated from mosquito cages at ~10–12 days post-emergence and their Malpighian tubules (MTs) were dissected out in physiological saline (25). Treatment conditions for female *Ae. aegypti* included 0.5hr and 24hr post blood meal (PBM), where ~10–15 females were blood fed at ~10–12 days old as described above. The females were allowed to feed for ~15min and then the time was initiated for each post-blood fed treatment with females that engorged on blood identifiable by their red abdomen. The rearing and treatments protocols were then kept consistent for each biological replicate.

### Immunohistochemistry

Immunohistochemistry was completed for whole body (WB) adult female *Ae. aegypti*, to localize the different AaAQPs. Immunohistochemistry was also completed for isolated adult female MTs from *Ae. aegypti*, localizing AaAQP1. For each treatment, NBF, 0.5hr PBM, 24hr PBM in WB and isolated MT sections, 4-5 individual mosquitoes (biological replicates) were studied. For each individual mosquito, 5–6 technical replicates were completed. Procedures were completed following previously published protocols (26–28). All tissues were fixed in Bouin’s fixative and dehydrated in a series of ethanol and xylene. Paraffin embedded tissues were then sectioned using an Epredia HM325 manual microtome (Epredia, Kalamazoo, Michigan United States) and placed on Fisherbrand^™^ ColorFrost^™^ Plus Adhesion Microscope slides. Samples were processed such that tissue sections from non-blood fed (NBF), 0.5hr PBM, and 24hr PBM mosquitoes were placed on the same slide. The slides were processed in a multi-day procedure, with stepwise washes in 1xPBS. WB tissue sections were probed with one of the following; Anti-AaAQP1 affinity-purified primary antibody (1:1000 rabbit polyclonal antibody against CFFKVRKGDEESYDF, Genscript, NJ, USA) (27), Anti-AaAQP2 affinity purified primary antibody (1:50 rabbit polyclonal antibody against CNGLGNTGLKENVQD, Genscript, NJ, USA) (29), Anti-AaAQP4 affinity purified primary antibody (1:500 rabbit polyclonal antibody against PAEQAPSDVGKSNQS, Genscript, NJ, USA) (27), Anti-AaAQP5 affinity purified primary antibody (1:1000 rabbit polyclonal antibody against FRREVPEPEYNRELT, Genscript, NJ, USA) (27), or Anti-AaAQP6 affinity purified primary antibody (1:50 rabbit polyclonal antibody against CSFRNMFLADKAKAE, Genscript, NJ, USA). WB tissue sections were also probed with a mouse monoclonal anti-a5 antibody for Na+/K+-ATPase (NKA) (Douglas Fambrough, Developmental Studies Hybridoma Bank, IA, USA, 1:10 dilution) as a membrane marker. Malpighian tubule tissue sections were probed with the same AaAQP1 antibody as previously listed, as well as a guinea pig anti-V_1_ antibody for V-type H^+^-ATPase (VA) (Ab 353-2, gifted by H. Wieczorek, Osnabruck, Germany, 1:5000 dilution) as a membrane marker, to specifically distinguish the apical membrane of the MTs. For secondary antibodies, a goat anti-rabbit AlexaFluor 594 (Jackson Immunoresearch) antibody was used (1:400 dilution) to visualize all of the AaAQPs, a goat anti-mouse secondary antibody conjugated to Cy2 (Jackson Immunoresearch) was used (1:500 dilution) to visualize NKA, and a goat anti-guinea pig AlexaFluor 488 (Jackson Immunoresearch) antibody (1:500 dilution) was used for VA. For control slides, the primary antibody was omitted to confirm an absence of staining where only secondary antibody was added. Similar controls were completed for all AaAQP primary antibodies used. Aside from omission of the primary antibody, control and experimental slides were treated identically. All samples were mounted on slides with ProLong^®^ Gold antifade reagent with DAPI (Life Technologies, Burlington, Ontario Canada). Slides were viewed and images captured using an Olympus IX81 fluorescent microscope (Olympus Canada, Richmond Hill, Ontario Canada) in combination with CellSense^®^ 1.12 Digital Imaging software (Olympus Canada). VA staining (AlexaFluor 488) and NKA staining (Cy2) were viewed using the Brightline GFP filter set and AaAQP staining (AlexaFluor 594) was viewed using the Brightline TRITC filter set (Olympus Canada). Exposure and gain settings were first determined by viewing sections from NBF mosquitoes and then the identical acquisition settings were used to view and capture images of the 0.5hr PBM and 24hr PBM mosquito sections all on the same slide. This same procedure was repeated for each slide of processed samples containing NBF and PBM mosquito sections. Identical acquisition settings were also used on control slides, to confirm that in the absence of primary antibody, no staining of AaAQPs was observed.

### Probe synthesis and fluorescence in-situ hybridization

Investigation into the identification and localization of *aqp1* mRNA began with *de novo* sequencing of the gene in *Ae. aegypti*. The originally reported transcript of *aqp1* by Pietrantonio et al. (20) was used as a template for our work, however it was discovered that annotations in the reference genome have reported different predicted transcripts variants of the *aqp1* gene, which ultimately yield different C-termini of the AaAQP1 protein. Through standard PCR and rapid amplification of cDNA ends (RACE), we confirmed the *aqp1* gene sequence (GenBank Accession: PP003259), which matches the originally reported sequence by Pietrantonio et al. (20) (see **Supplementary Materials and Methods** and **Supplementary Results**). We also used heterologous expression in human embryonic kidney (HEK293T) cells to verify our custom antibody against AaAQP1 specifically detects this water channel and the immunoblot results demonstrate a band size that matches that observed in protein extracted from MTs (**Supplementary Figure S2**).

To synthesize a template suitable for fluorescence *in situ* hybridization (FISH) with DIG-labelled RNA probes, *aqp1* FISH forward and reverse primers (**Supplementary Table S1**) were used to amplify a 583bp *aqp1* fragment. Primers were designed using the Primer3 plugin in Geneious® 8.1.8 (Biomatters Ltd., Auckland, New Zealand). The *aqp1* gene was then amplified using bacterial cloning, by ligating the product into the pGEM T-Easy cloning vector (Promega, Madison, Wisconsin, USA) and the previously described standard protocol for cloning (12) was followed to amplify the specific portion of the *aqp1* gene. During colony screening, *aqp1* FISH primers with added T7 promoter sequences were used to yield *aqp1* gene fragments with added T7 sequences for RNA probe synthesis. Confirmation of fragment size was done by running an agarose gel and colonies yielding PCR products with correct size were chosen for overnight inoculation and then plasmid DNA was isolated by standard column-based plasmid mini-prep (Bio Basic Inc., Markham, Ontario, Canada), according to the protocol associated with the kit. Products were diluted 1:100 and ran on an agarose gel to confirm product size and band intensity. Then several replicate PCR reactions were completed to generate a large volume of both the anti-sense and sense DNA template products used for preparing RNA probes for experimental and control preparations, respectively. Products were pooled and purified using the Monarch PCR and DNA Cleanup Kit (5µg). The DNA template concentrations were determined using the Synergy Multi-Mode Microplate Reader (BioTek, Winooski, USA). To synthesize the digoxigenin (DIG) labeled RNA probes, the purified DNA products (either anti-sense or sense template) were added to a PCR tube with reaction buffer, a T7 polymerase, and DIG RNA Labeling Mix (Roche Applied Science, Mannheim, Germany), following the T7 RNA Polymerase Kit (New England BioLabs, Ontario, Canada). The tubes were incubated overnight at 37°C and the following day, the probe products were diluted 1:10 and were treated with a DNAse I to remove the DNA template. Products were run on a RNase-free non-denaturing agarose gel to confirm RNA probe size and band intensity.

To prepare tissue samples for FISH, we followed a protocol previously described (30). First, MTs were dissected from 3–4 day old adult female *Ae. aegypti* and transferred into a microcentrifuge tube containing 200µL of sterile PBS. The PBS was then replaced with freshly prepared 4% paraformaldehyde (PFA) and placed on a rotator for 1hr at room temperature to fix the tissues. The PFA was removed and tissues were washed five times with sterile PBT (sterile PBS + 0.1% Tween-20). Tissues were then quenched with 1% H_2_O_2_ for 20 min at room temperature to quench endogenous peroxidase activity which would otherwise result in elevated non-specific background fluorescence. Following this, tissue samples underwent permeabilization using 4% Triton X (960µl PBT + 40µL Triton X-100) with tubes set on a rocker at room temperature for 1hr. Tissues were then washed three times with PBT and followed by a second fixation with 4% PFA, with tissues rotating at room temperature for 20 min. The MTs were then rinsed with 1:1 PBT : Hybridization solution (Hyb) (50% formamide, 5x SSC, 100ug/mL heparin, 100ug/mL sonicated salmon sperm DNA and 0.1% Tween-20), followed by a single wash with 100% Hyb. All subsequent incubations above or below room temperature were carried out on a thermocycler. Pre-Hyb solution was made during this time by aliquoting 250µL per sample of Hyb solution into PCR tubes which were then placed at 100°C for 5 min, followed by a 5 min incubation on ice. After removal of 100% Hyb from the sample tubes, pre-Hyb solution was added to the sample and tissues were incubated at 56°C for 1hr. During this time, 4ng/µL of anti-sense (experimental) or sense (control) probe was prepared in pre-Hyb solution and then applied to the tissues overnight at 50°C. Solutions for the following day were prepared at this time, including 100% Hyb, 3:1 Hyb : PBT, 1:1 Hyb : PBT, 1:3 Hyb : PBT, and 100% PBT, all incubated overnight at 50°C.

The next day, tissues were washed in the following 50°C pre-warmed solutions; twice with 100% Hyb, once with 3:1 Hyb : PBT, once with 1:1 Hyb : PBT, once with 1:3 Hyb : PBT, and once with 100% PBT. Then, blocking of tissues to reduce non-specific staining was done with 1% blocking solution, PBTB (0.1g Molecular Probes block reagent; Invitrogen, Carlsbad, USA + 9.5mL PBT), rotating for 1hr at room temperature. The tissues were then incubated for 1.5hr with 1:200 mouse anti-DIG biotin-conjugated antibody (Jackson Immuno Research, West Grove, USA) diluted in PBTB, rotating at room temperature. From this step and onward, tissue samples were protected from light exposure. Tissues were then washed with PBTB four times, for 15 min each, rotating at room temperature. The tissue samples were then incubated with 1:50 HRP-streptavidin in PBTB for 1hr, rotating at room temperature to bind with the biotin-conjugated anti-DIG primary antibody. Tissues were once again washed with PBTB four times, for 15 min each, rotating at room temperature. Following manufacturer instructions, the samples were then incubated in 100µL diluted tyramide solution (Life Technologies, Eugene, USA) for 5 min at room temperature and then immediately followed by the addition of 100µL of stop solution (Life Technologies, Eugene, USA). The solution was then removed, and ten PBS washes were completed, following by an overnight incubation with PBS, rotating at room temperature and protected from light. Tissue samples were mounted on slides the following day using in-house mounting media (1:1 PBS:glycerol containing 4µg/mL DAPI) and were imaged with the EVOS FL Auto Live-Cell Imaging System (Life Technologies, Burlington, ON). The fluorescence *in situ* hybridization experiments were completed in at least four biological replicates that each included experimental (anti-sense probe) and control (sense probe) preparations. Image acquisition settings were identical for all sample preparations including experimental samples treated with anti-sense probes and control samples treated with sense-probes.

### Transmission electron microscopy and immunogold staining

Transmission electron microscopy (TEM) techniques and imaging was carried out by Dr. Ali Darbandi at the Nanoscale Biomedical Imaging Facility at The Hospital for Sick Children Research Institute – Peter Gilgan Centre for Research and Learning. The MTs from non-blood fed adult female *Ae. aegypti* were dissected in physiological saline (25) and fixed in 2% paraformaldehyde and 0.5% glutaraldehyde in 0.1M sodium cacodylate for 2hr at room temperature. The MTs were then rinsed in buffer and dehydrated in a graded ethanol series (50%, 70%, 90%, and 100%) for 20 min each at 4°C. Following this, two 1:1 ethanol/LR white acrylic resin changes were made for 30 min each and then tissue samples were embedded in LR white resin where blocks were left to cure overnight at 60°C. MT sections of 70nm thickness were cut on a Leica EM UC7 ultramicrotome (Ontario, Canada) and the sections were stained with uranyl acetate and lead citrate. The grids were imaged using an electron microscope at the Nanoscale Biomedical Imaging Facility at The Hospital for Sick Children Research Institute – Peter Gilgan Centre for Research and Learning.

For immunogold labeling, MT samples were prepared on TEM grids as previously described by Schwartzbach and Osafune (31), completed at the Peter Giligan Centre for Research and Learning, Toronto CA. Then, each individual grid was placed on a 100µl droplet of 0.01M sodium citrate at 95°C for 15 min, followed by a brief 5 min cooling period and two washes with 0.15M glycine for 15 min and 1xPBS for 5 min. The grids were placed on individual 100µl droplets of antibody dilution buffer (ADB) blocking for 30 min at room temperature, followed by three washes with 1xPBS, 1% BSA, and 0.05% Tween^®^20 (Bio-Rad) for 5 min each and a 2hr wash in 1x PBS, 10% BSA, and 0.05% Tween^®^20 (Bio-Rad). The tissues were probed with 1:5 anti-AaAQP1 affinity purified rabbit polyclonal antibody in 1x PBS, 10% BSA, and 0.05% Tween^®^20 (Bio-Rad) overnight at 4°C. The grids were washed five times in 1x PBS with 1% BSA for 5 min each, followed by a 1hr incubation in a colloidal gold AffiniPure goat anti-rabbit secondary antibody conjugated with 18nm gold particles (Jackson Immunoresearch) in 1% BSA and 0.05% Tween^®^20 (1:10) (Bio-Rad). For control samples, they were treated identical to the experimental grids previous to this step. However, for control grids the primary anti-AaAQP1 antibody was omitted, to show its specificity relative to the treated samples. All tissue samples (control and experimental) were then treated with 2% glutaraldehyde in PBS for 5 min, before a series of washes with ddH_2_O and the grids were stained with uranyl acetate and lead citrate. Imaging was completed at the same facility using the electron microscope.

### Gel electrophoresis and western blotting

Gel electrophoresis and western blotting was completed on protein samples isolated from adult female *Ae. aegypti* MTs. For each biological replicate (n=5–6), MTs were collected from 75 individual female mosquitoes under physiological saline (25), from NBF, 0.5hr PBM, and 24hr PBM groups. Protein processing for all samples was done using the Mem-PER Plus Membrane Protein Extraction kit (Thermo Fisher Scientific, Burlington, Ontario Canada) as recently described (32). First, the cytosolic fraction of the MT protein was separated by adding 60µl of permeabilization buffer from the kit to each sample tube, with 1:200 protease inhibitor cocktail (Thermo Fisher Scientific, Burlington, Ontario Canada). The tubes were set to mix on a rotator at 4°C for 10min, before centrifugation at 16,000g for 15min at 4°C. The supernatant was collected as the cytosolic fraction. The remaining pellet was then re-suspended in 60µl of solubilization buffer with 1:200 protease inhibitor cocktail (as above) and set to incubate at 4°C for 30min. The samples were then centrifuged at 16,000g for 15min at 4°C and the supernatant was collected as the membrane fraction. Protein concentrations were measured using a Bradford assay, relative to bovine serum albumin (BSA) standards.

The MTs samples were prepared for SDS-PAGE, where MT protein was combined with 50mM Tris buffer and 6x loading buffer [225mmol L^−1^ Tris-HCl, pH 6.8, 3.5% SDS, 35% glycerol, 12.5% ß-mercaptoethanol, and 0.01% bromophenol blue]. The samples were then heated for 5 min at 100°C, placed back on ice briefly, and centrifuged at 10,000g for 1 min. For each replicate, 5µg of protein were loaded in 12% SDS-PAGE gels. Following gel electrophoresis at 120V, a wet transfer was completed where MT protein was transferred onto a polyvinyl difluoride membrane at 90V for 2hr on ice in 1x transfer buffer (0.225g Tris, 1.05g glycine, 20% methanol in 1L of ddH_2_O). Following the transfer, the membranes were placed in 5% milk blocking buffer (5g skim milk powder, 100mL 1x Tris-buffered saline [TBS-T; 0.12g Tris, 0.9g NaCl, 0.1mL Tween^®^20 (Bio-Rad), 0.1mL NP-40 (Sigma-Aldrich) in 1L of ddH_2_O]) at room temperature for 1hr on a rocker. The membranes were then probed with 1:1000 specific anti-AaAQP1 affinity-purified primary antibody (1.282 μg/mL) (27) overnight at 4^°^C and then washed in 1x TBS-T the following day before incubation in horseradish peroxidase (HRP)-conjugated goat anti-rabbit antibody at 1:5000 in blocking buffer for 1hr at room temperature. After a second series of washes with 1x TBS-T, 2mL of prepared chemiluminescent Clarity™ Western ECL substrate kit (BioRad, Hercules, California, United States) was applied to each membrane for 5 mins. The membranes were viewed using a Chemi-Doc MP Imaging System (BioRad, Hercules, California, United States) and protein bands were normalized against Coomassie staining of total protein. The protein bands were analyzed using ImageJ 1.53a Software (USA) to quantify protein abundance, normalized to total protein.

### Statistics

All data was analyzed using Prism^®^ 9 software (GraphPad Software Inc., California, USA). Normalized protein abundance values were plotted as mean values ± standard error of the mean (SEM). For *Ae. aegypti* western blot graphs, data was normalized to non-blood fed control groups. An unpaired t-test was completed for each data set to determine if there were significant changes in AaAQP abundance values. The ROUT outlier test was used to determine if there were outlier values, which were removed accordingly.

## Results

### Localization of AaAQPs in adult female *Ae. aegypti*

AaAQP1, 2, 4, 5, and 6 were localized in whole body (WB) tissue sections of adult female *Ae. aegypti* mosquitoes with Na^+^/K^+^-ATPase (NKA) used as a membrane marker.

### AaAQP1

**Figure 1:**
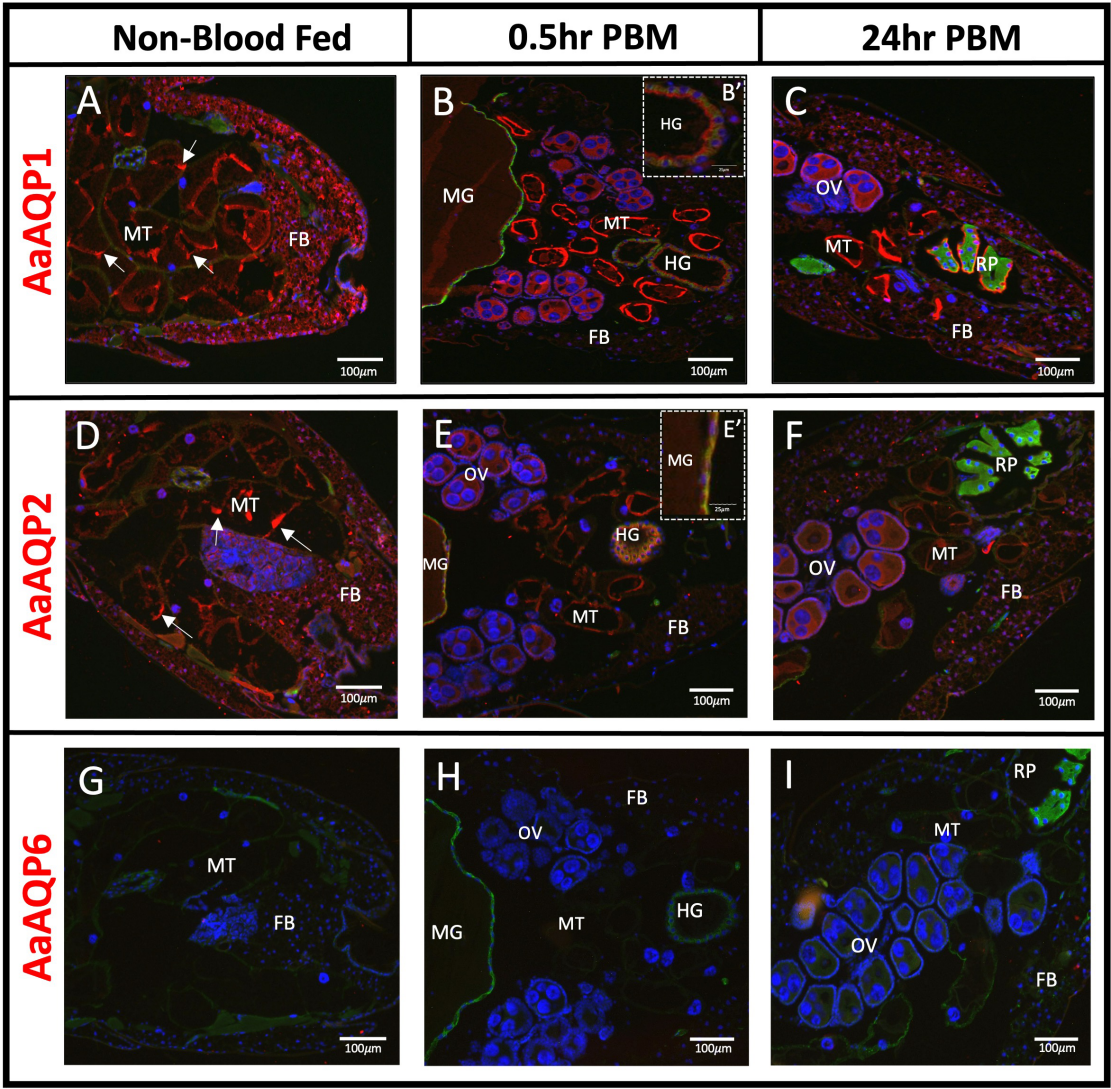
Localization of AaAQP1, 2, and 6 in whole body adult female *Ae. aegypti.* Immunohistochemical localization of water selective AaAQPs (red staining) in female *Ae. aegypti* mosquitoes (~10–12 days old). Each AaAQP (red staining) was localized under non-blood fed conditions, 0.5hr post blood meal conditions, and 24hr post blood meal conditions. Whole body sections of the abdominal segment immunolocalizing AaAQP1 in **(A–C)**, AaAQP2 in **(D–F)**, and AaAQP6 in **(G–I)**. In **(B)**, an inset image **(B’)** shows AaAQP1 membrane localization in the HG. In **(E)**, an inset image **(E’)** shows AaAQP2 membrane localization in the MG. All images were taken at 10x magnification (n=3). The Na^+^/K^+^-ATPase (NKA) was used as a membrane marker (green staining) and nuclei were stained with DAPI (blue). MT, Malpighian tubules; FB, fat body; OV, ovaries; HG, hindgut; MG, midgut; RP, rectal pads. White arrows indicate aggregated staining of AaAQP in the MTs.

AaAQP1 immunoreactivity was primarily detected in the Malpighian tubules (MTs) and the fat body (FB), under non-blood fed (NBF) conditions (**Figure 1A**). AaAQP1 immunoreactive staining in the MTs appears aggregated in some areas but, for the most part, is evenly distributed at the apical side of the cell, in the NBF conditions (**Figure 1A**). At 0.5hr post blood meal (PBM), staining of AaAQP1 appeared uniform at the apical membrane of the MTs, with stronger staining intensity compared to NBF mosquitoes (**Figure 1B**). At 24hr PBM, immunoreactive staining of AaAQP1 is similar to the 0.5hr PBM in the MTs (**Figure 1C**). AaAQP1 staining in the fat body appeared to decrease in intensity at 0.5hr PBM in comparison to the NBF group (**Figure 1B**). However, at 24hr PBM, staining intensity of AaAQP1 in the fat body appeared more intense in comparison to the 0.5hr PBM group, but less intense compared to the NBF condition (**Figure 1C**). Interestingly, prominent AaAQP1 immunoreactive staining was localized to the ovaries in both the 0.5hr and 24hr PBM groups (**Figures 1B, C**). Minimal immunoreactivity of AaAQP1 was detected in midgut sections visible in the 0.5hr PBM group (**Figure 1B**). Furthermore, AaAQP1 immunoreactivity was found in the apical membrane of the hindgut in the 0.5hr PBM group and as well as on the apical side of the cells in the rectal pads in the 24hr PBM group, showing intense staining (**Figures 1B, B'**, **C**).

### AaAQP2

Immunoreactivity for AaAQP2 was detected in the MTs, showing dispersed and apical membrane staining in the NBF group (**Figure 1D**). In both the 0.5hr and 24hr PBM groups, the overall staining intensity of AaAQP2 immunoreactivity remains the same but staining appears uniformly at the apical membrane, in comparison to the NBF group (**Figure 1E**). Immunoreactivity of AaAQP2 appeared intense in the fat body under NBF conditions, with a reduction in intensity in the 0.5hr PBM group, and a partial recovery of staining intensity in the 24hr PBM group (**Figures 1D–F**). Further, AaAQP2 immunoreactivity was found in the ovaries of the blood fed groups. Finally, AaAQP2 immunoreactive staining was also present in the midgut and hindgut tissue in the 0.5hr PBM, where it was co-localized with the NKA membrane marker appearing at the basolateral membrane (**Figure 1E, E'**).

### AaAQP6

Mosquito abdominal sections were void of any AaAQP6 immunoreactive staining in NBF and PBM conditions (**Figures 1G–I**).

### AaAQP4

**Figure 2:**
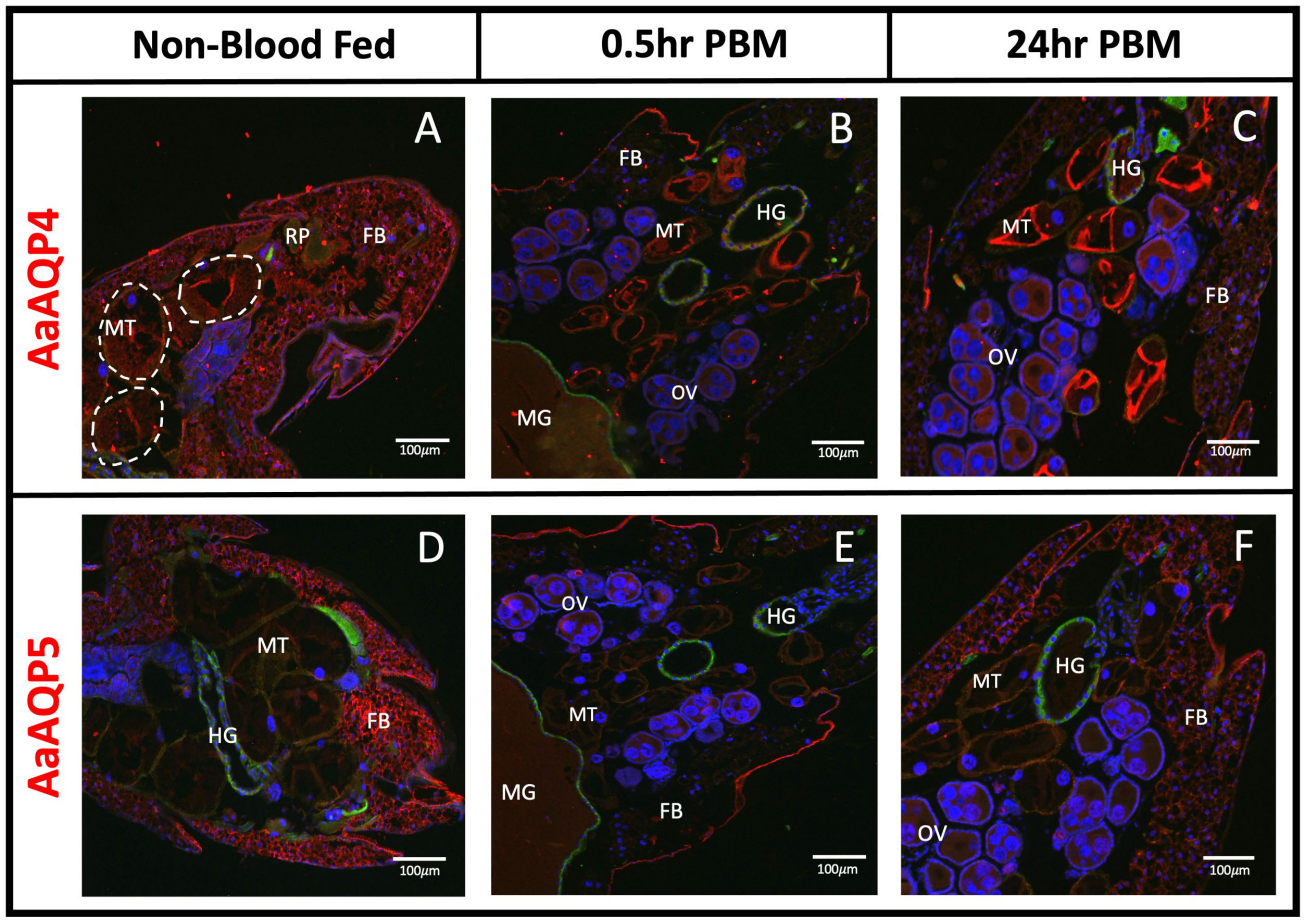
Localization of AaAQP4 and 5 in whole body adult female *Ae. aegypti.* Immunohistochemical localization of entomoglyceroporin AaAQPs (red staining) in female *Ae. aegypti* mosquitoes (~10–12 days old). AaAQP4 and 5 (red staining) were localized under non-blood fed conditions, 0.5hr post blood meal conditions, and 24hr post blood meal conditions. Whole body sections of the abdominal segment immunolocalizing AaAQP4 in **(A–C)** with basolateral membrane of MTs encircled in dashed line and AaAQP5 in **(D–F)**. All images were taken at 10x magnification (n=3). The Na^+^/K^+^-ATPase (NKA) was used as a membrane marker (green staining) and nuclei were stained with DAPI (blue). MT, Malpighian tubules; FB, fat body; OV, ovaries; HG, hindgut; MG, midgut; RP, rectal pads. White arrows indicate aggregated staining of AaAQP in the MTs.

Immunoreactivity for AaAQP4 was detected in the MTs, under all three conditions (**Figures 2A–C**). Staining appeared at the apical membrane of the MTs, with similar intensity seen in the NBF and 0.5hr PBM groups, but an increase in staining intensity was observed in the MTs in the 24hr PBM group (**Figure 2C**). Immunoreactivity for AaAQP4 was also found in the fat body, appearing intense in the NBF group, reduced in the 0.5hr PBM group, and partially recovered in the 24hr PBM group (**Figures 2A–C**). AaAQP4 was also found in the hindgut of 0.5hr and 24hr PBM tissue sections, with relatively low immunofluorescence (**Figures 2B, C**). Additionally, minimal immunoreactivity was observed in the ovaries at 0.5hr and 24hr PBM (**Figures 2B, C**).

### AaAQP5

Immunoreactivity for AaAQP5 in NBF mosquitoes appeared relatively low and uniformly distributed at both the apical and basolateral membranes of the MTs (**Figure 2D**). AaAQP5 immunoreactive staining was found mainly in the fat body in NBF conditions; however, staining was absent in the fat body in the 0.5hr PBM but was localized to the epidermis of the cuticle (**Figure 2E**). At 0.5hr PBM, there was some localized staining seen in the ovaries (**Figure 2E**). At 24hr PBM, AaAQP5 immunoreactive staining in the fat body returned (**Figure 2F**) with no additional staining observed in the midgut, ovaries and hindgut.

### AaAQP1 is expressed by principal cells in the MTs of adult female *Ae. aegypti*

**Figure 3:**
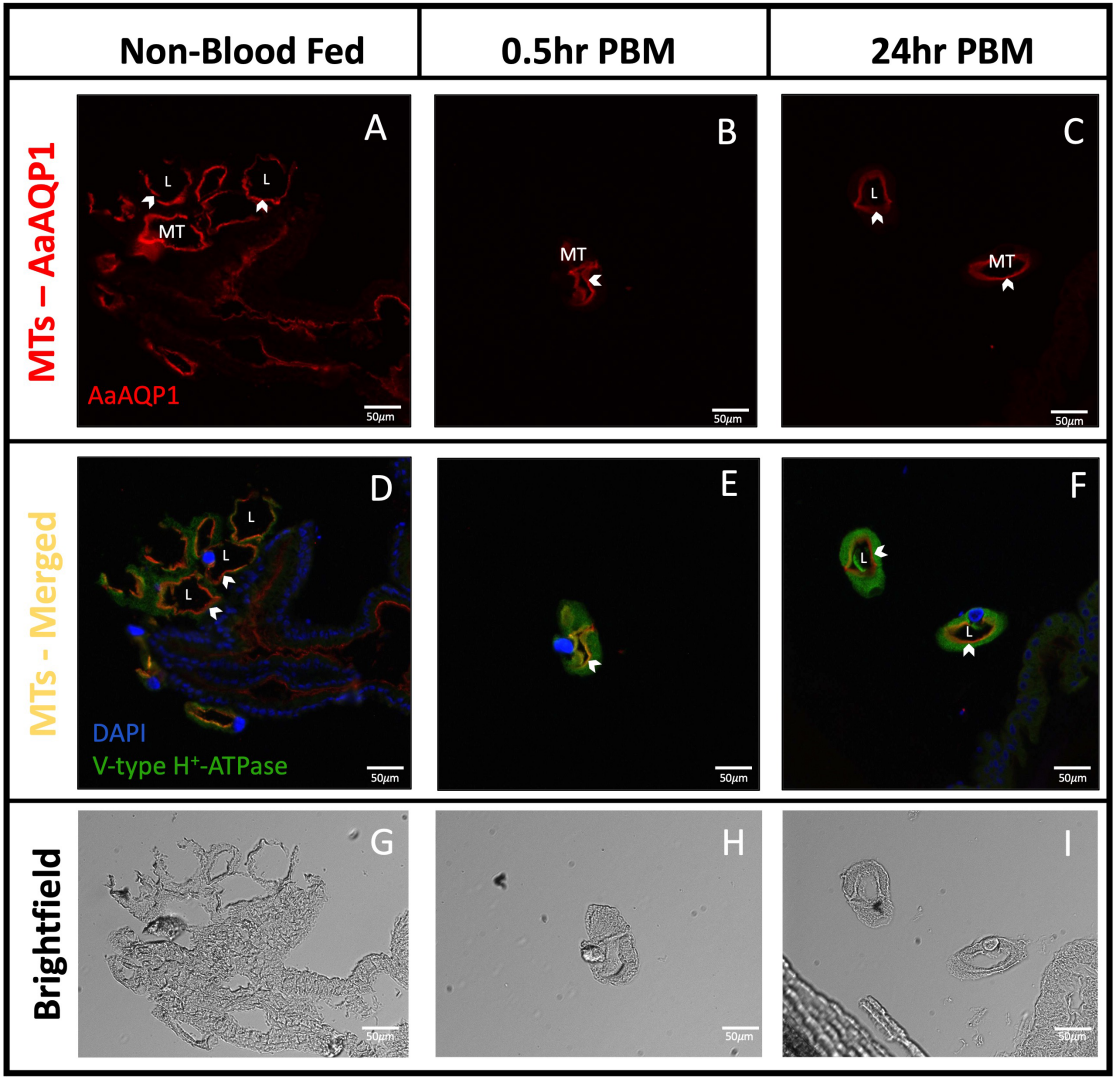
Localization of AaAQP1 in Malpighian tubules of adult female *Ae. aegypti.* Immunohistochemical localization of AaAQP1 (red staining) in isolated Malpighian tubules (MTs) of female *Ae. aegypti* (~10–12 days old) MTs were isolated from non-blood fed mosquitoes, and mosquitoes that were fed blood either 0.5hr or 24hr prior. **(A–C)** shows localization of AaAQP1 at the apical membrane of the MTs. White arrows indicate the apical membrane of the MTs. **(D–F)** shows localization of AaAQP1 at the apical membrane of the MTs, merged with staining observed for V-type H^+^-ATPase (VA), which was used as an apical membrane marker (green staining). Co-localization of AaAQP1 with VA appears yellow/orange in colour. **(G–I)** shows brightfield images of MT sections, which was used to identify apical and basolateral membranes. All images were taken at 20x magnification (n=4) and nuclei were stained with DAPI (blue).

**Figure 4:**
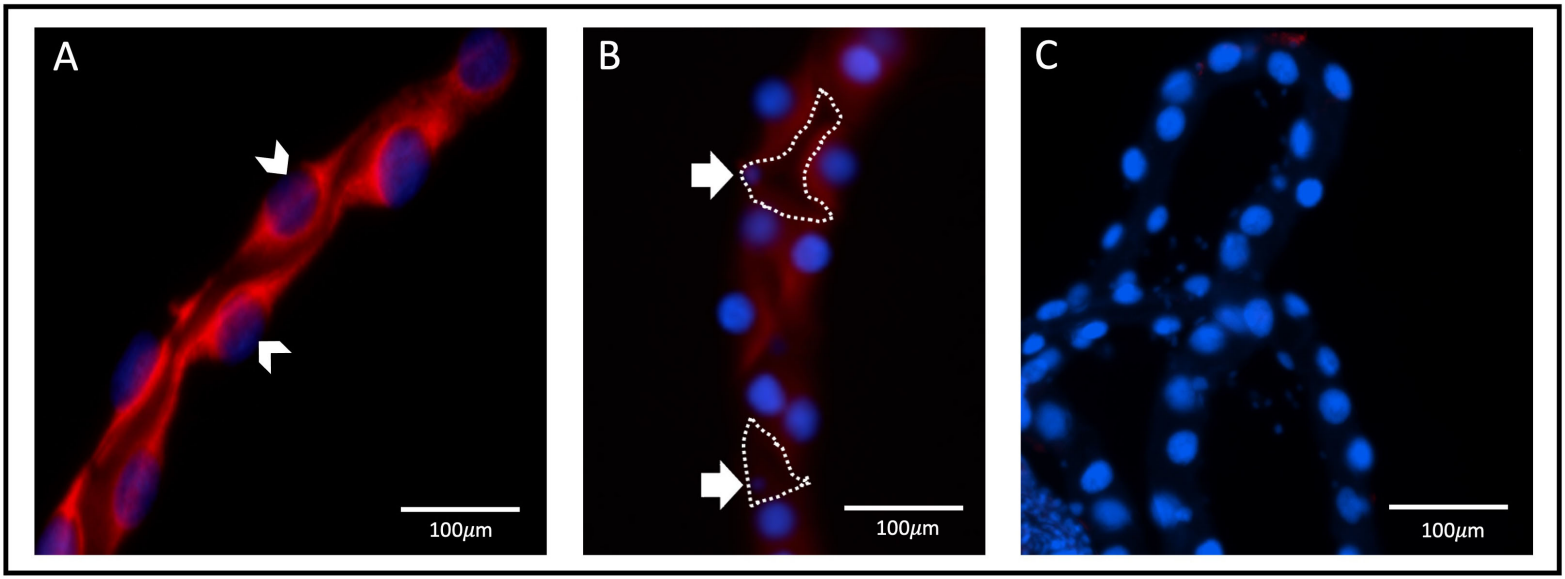
*aqp1* localization with fluorescence *in situ* hybridization in Malpighian tubules. Localization of *aqp1* mRNA (red staining) in the Malpighian tubules (MTs) of female *Ae. aegypti* mosquitoes (~3–4 days old), using fluorescence *in situ* hybridization. Tubules were dissected from non-blood fed adult female mosquitoes (n=3). **(A)** *aqp1* localization in the distal tubule, found associated with the principal cell nuclei, indicated by the white arrow. **(B)** *aqp1* localization in the proximal tubule, found associated with the principal cells. Stellate cell nuclei indicated by the white arrow, with cell structure outlined in dashed lines, showing an absence of staining in encircled region. **(C)** MTs where an absence of *aqp1* staining is seen, after incubation and treatment with the sense (control) probe.

Sections of isolated MTs confirmed that AaAQP1 staining is present on the apical membrane of the epithelia (**Figures 3A–I**), where co-localization with the V-type H^+^-ATPase (VA) can be observed in the 0.5hr PBM mosquito tubules (**Figure 3E**). Importantly, *in situ* hybridization using an anti-sense probe demonstrated that *aqp1* mRNA in adult female *Ae. aegypti* MTs was associated with the larger and more abundant principal cells. In the proximal tubule, *aqp1* transcript was localized in the cytosol around the nuclei of principal cells (**Figure 4A**) while *aqp1* transcript staining was absent in stellate cells (**Figure 4B**). In the distal portion of the adult female tubule, very strong *aqp1* transcript levels were observed with consistent and exclusive staining to the principal cells of the MTs (**Figures 4A, B**). No staining was observed in MTs treated with the sense probe (**Figure 4C**), confirming the localization of *aqp1* transcript detected with the anti-sense probe (**Figures 4A, B**).

### Sub-cellular localization of AaAQP1 in MTs of adult female *Ae. aegypti*

**Figure 5:**
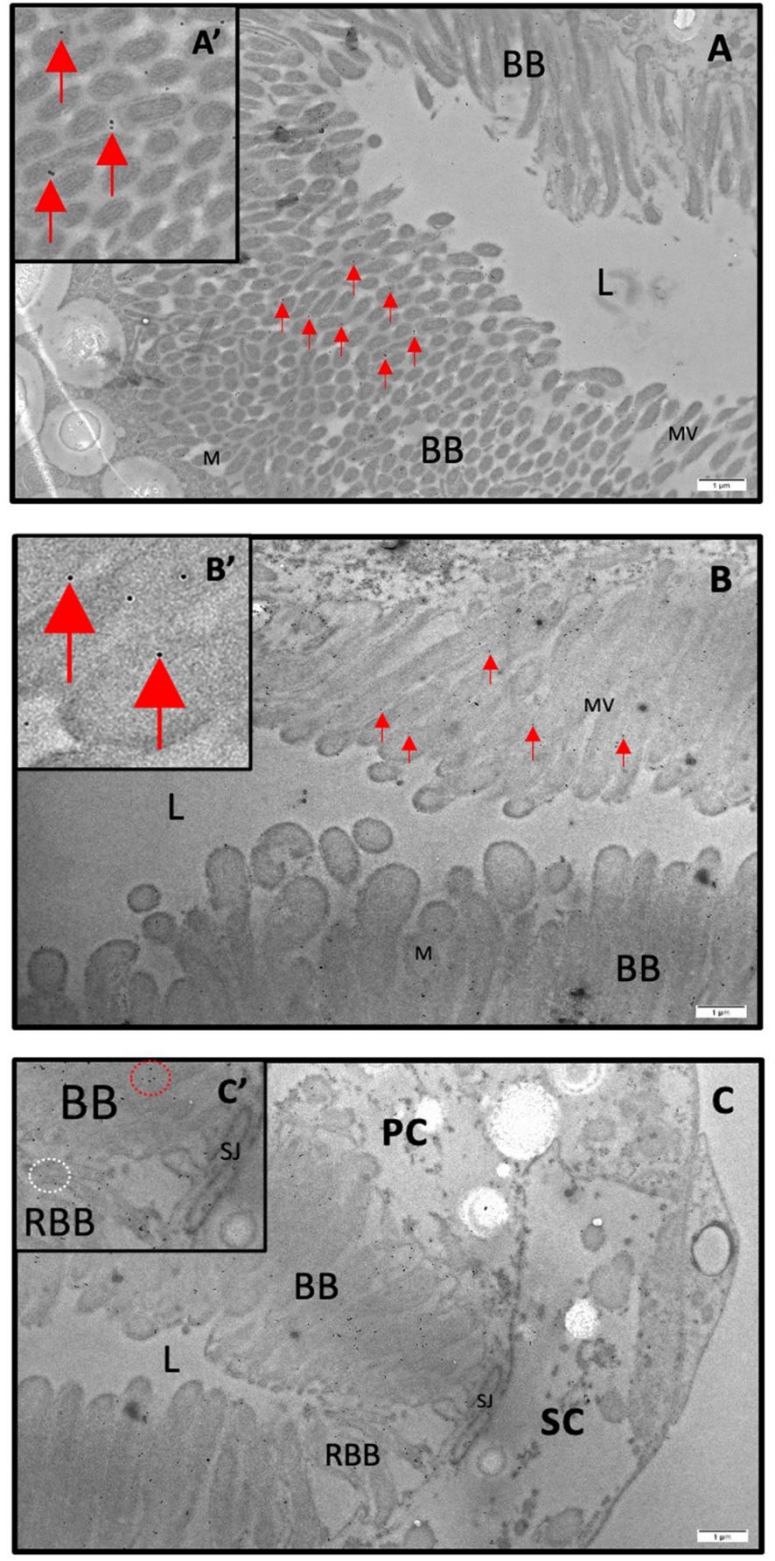
Immunogold staining of AaQP1 in female Malpighian tubules of *Ae. aegypti.* Subcellular localization of AaAQP1 in adult female *Ae. aegypti* (~10–12 days old) Malpighian tubules, using transmission electron microscopy (TEM) with immunogold staining (n=3). AaAQP1 staining is represented by the presence of 18nm gold particles, indicated by the red arrows. Inset images magnify individual gold particles. **(A, B)** Cross sections of non-blood fed mosquito Malpighian tubules, showing the apical membrane of the principal cells. Gold particles appear dispersed through the membrane fraction specifically within the brush border, with no pattern of gold particle collection. **(C)** Cross section of non-blood fed mosquito Malpighian tubules showing predominant gold particle presence in principal cells; however, there is some presence of gold particles also within the reduced brush border of the stellate cells. BB, brush boarder; L, lumen; M, mitochondria; MV, microvilli; RBB, reduced brush boarder.

Immunogold labeling and transmission electron microscopy were used to localize AaAQP1 at the sub-cellular level in MTs of adult female mosquitoes. In NBF MTs, immunogold particles were predominantly localized to the principal cells (PCs) and appear scattered throughout the apical region in the brush border (**Figures 5A, B**). Additionally, some immunogold particles were also detected in reduced brush border of the less abundant stellate cells (SCs) (**Figure 5C**). Control grids, where the primary antibody was omitted, showed a complete absence of gold particles, relative to the presence of gold particles in the sections treated with both primary and secondary antibodies (**Supplementary Figure S1**).

### Quantitative assessment of AaAQP1 protein expression in MTs of adult female *Ae. aegypti*

**Figure 6:**
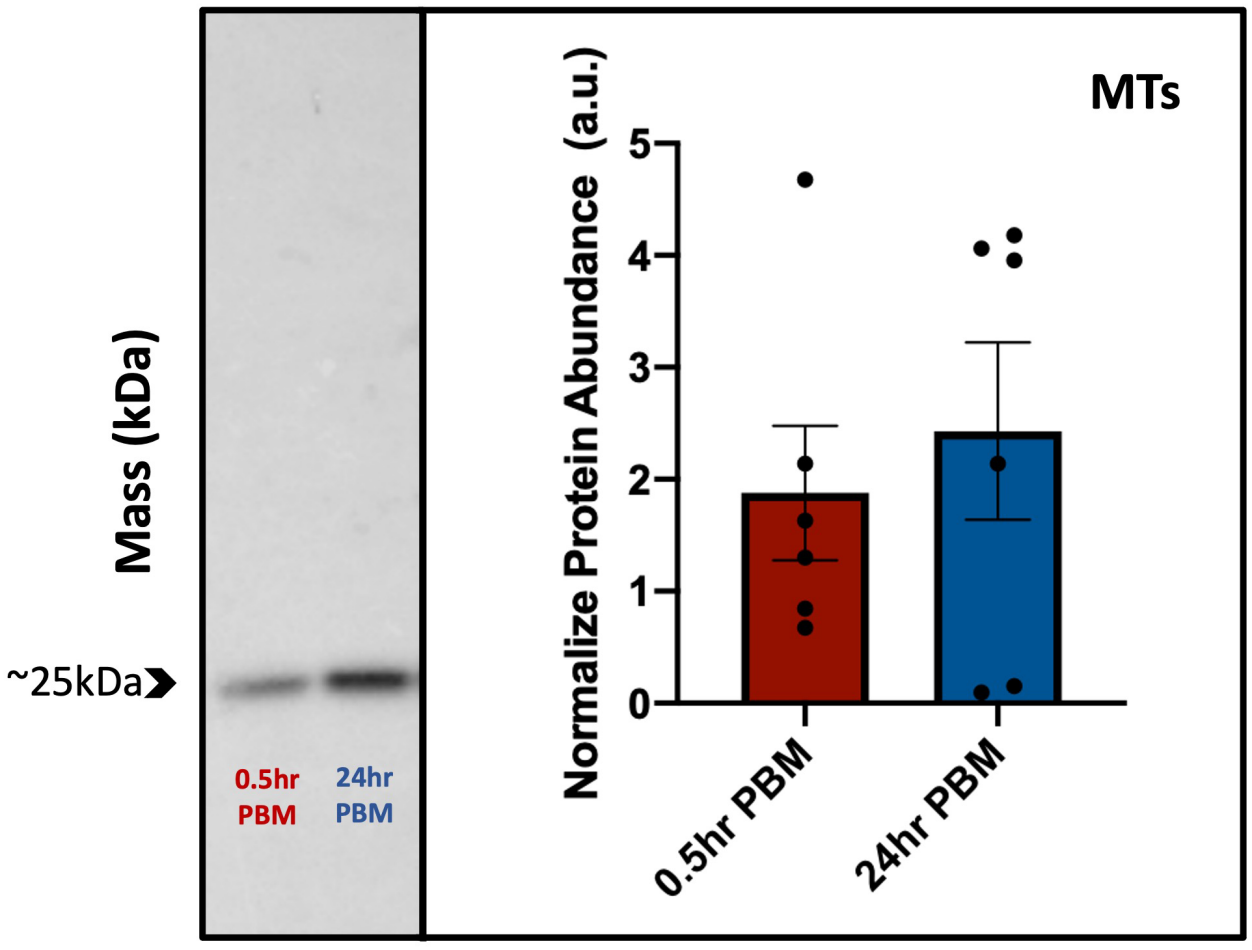
Protein abundance of AaAQP1 in female *Ae. aegypti* Malpighian tubules post blood meal. Normalized protein abundance of AaAQP1 in female *Ae. aegypti* Malpighian tubule tissue samples, in 0.5hr post-blood meal and 24hr post-blood meal groups. Values were normalized to the AaAQP1 immunoreactive band detected in Malpighian tubule protein samples from non-blood fed control mosquitoes. Malpighian tubules were isolated from ~75 individuals, for each biological replicate (n=5–6). A ~25kDa band (putative monomer) was detected for AaAQP1, and an unpaired t-test showed no significant changes in the protein abundance between the two blood fed treatment groups (p>0.05).

A ~25kDa band representing the putative monomer for AaAQP1 was observed in western blots of protein homogenates from MTs of adult female *Ae. aegypti* probed with custom anti-AaAQP1 antibody (**Figure 6**). Blood feeding did not result in any changes in AaAQP1 protein abundance in the MTs. There were no significant differences between AaAQP1 levels in MTs of the 0.5hr PBM and 24hr PBM groups, normalized to the NBF control. Therefore, there were no differences found in AaAQP1 protein in post blood fed female MTs relative to the NBF control. Heterologous expression of the AaAQP1 protein in human embryonic kidney cells yielded a protein of identical size, ~25kDa, confirming the specificity of the custom antibody utilized in this study (**Supplementary Figure S2**).

## Discussion

In this study, we immunolocalized the aquaporins AaAQP1, 2, 4, 5, and 6 in organs of the adult female mosquito, *Ae. aegypti*, before and after a blood meal. Furthermore, a primary aim of this study was to better understand the function of the primary water channel, AaAQP1, in the adult female *Ae. aegypti* mosquito. For this reason, we focused more extensively on AaAQP1 expression at both the protein and transcript levels in the Malpighian tubules since earlier studies have shown its mRNA is enriched in this organ (16).

### AaAQP localization in the fat body

The transcript expression of AaAQPs in the fat body was previously reported where relatively low levels of *aqp1–6* mRNA were found (16, 33). Our data demonstrated immunolocalization of AaAQP1 (**Figures 1A–C**), 2 (**Figures 1D–F**), 4 (**Figures 2A–C**) and 5 (**Figure 2D–F**) in the fat body, but did not observe AaAQP6 immunoreactive staining in this tissue (**Figure 1G–I**). The intensity of AaAQP1, 2, 4, and 5 immunoreactive staining in the fat body decreased at 0.5hr PBM compared to NBF suggesting that blood feeding leads to lowered expression of these aquaporins in the fat body. Using transcriptomic analysis, Price at al. (33) detected three of the six mosquito aquaporins expressed in the fat body, including AaAQP1, 4, and 5 (**Table 1**). Notably, Price et al. reported an apparent decrease in the transcript abundance of AaAQP4 and AaAQP5 in the fat body 24 hours after a blood meal (33). Our results are consistent with this data and furthermore suggest that there is an abrupt decrease in AaAQP4 and AaAQP5 protein expression after a blood meal (**Figure 2**). It has been hypothesized that a decrease in overall transporters PBM in *Ae. aegypti*, including AaAQP4 and 5, may be accounted for by the increase in transporters present in yolk proteins PBM, during embryogenesis (33). On the other hand, AaAQP1 transcript was not detected in NBF fat body although low levels were detectable in this tissue at 24hr PBM (33), which is consistent with our observations of the partial recovery of AaAQP1 immunoreactive staining at 24hr PBM (**Figure 1C**). In addition, the expression of an AaAQP2 ortholog, AgAQP1 in the malaria vector, *Anopheles gambiae*, found a significant increase in the fat body, 48hr PBM (34). A similar trend in the expression of AaAQP2 is apparent in the fat body where staining intensity is largely diminished at 0.5hr PBM followed by partial recovery at 24hr PBM (**Figures 1D–F**). These findings suggest that blood feeding may lead to a temporary short-term reduction (~0.5hr PBM) in AaAQP1 and AaAQP2 expression in the fat body of mosquitoes but, the expression of these two aquaporins partially recovers within a day or two of blood feeding. It is possible that AaAQP1 and 2 proteins are more immediately relevant for the MTs or HG, during post-prandial diuresis and early-stage blood meal digestion when the animal is dealing with a large load of water associated with the 0.5hr PBM female mosquitoes. During late-stage blood meal digestion, such as 24hr PBM, increased AaAQP1 and 2 staining in the fat body might relate to roles in water transport during egg maturation.

The fat body is an important multifunctional organ for female mosquitoes participating in nutrient storage, metabolic homeostasis, and production of yolk precursor proteins for egg production (35). In the previtellogenic female mosquito that has not blood fed, the fat body stores nutrients, and the trophocytes (i.e. fat body cells) contain numerous, relatively large lipid droplets which are synthesized at the endoplasmic reticulum (33, 36, 37). The fat body trophocytes are activated by a blood meal, shifting their function into yolk precursor protein (YPP) factories, which includes vitellogenin and lipophorin that are secreted into the haemolymph where they are then taken up by developing oocytes in the ovaries (33, 36, 37). During this time of YPP synthesis, the size of lipid droplets fluctuates, but by 24hrs PBM, they are reduced in size and by 48hrs PBM, they have recovered to pre-blood meal sizes (37). Furthermore, by 36hrs PBM, the fat body trophocytes revert back to a nutrient storage organ (36). Abundant immunoreactive staining of AaAQP4 and 5 correlates to the nutrient storage stage of the fat body trophocytes where lipid synthesis and storage are likely occurring at higher rates. When trophocytes are activated to synthesize YPPs for secretion into the haemolymph, AaAQP4 and 5 immunoreactive staining is low. Since AaAQP4 and 5 are entomoglyceroporins which have been shown to transport glycerol in a heterologous system, their function in the trophocytes may be to facilitate glycerol transport for the synthesis of lipids when the main function of the fat body is nutrient storage (13). AaAQP1 and 2 are the orthologs of *Drosophila* DRIP and PRIP, respectively and both have been shown to exhibit a preference for water transport (13, 16). The intensity of AaAQP1 and AaAQP2 immunoreactive staining suggests that similar to the entomoglyceroporins, their most abundant expression in the fat body coincides with nutrient storage and metabolic function of the trophocytes. Since lipid metabolism requires water, these aquaporins may play a critical role in regulating water content of the trophocytes as they synthesize fats. Comparatively, diminished AaAQP1 and 2 immunoreactive staining coincides with when trophocytes are synthesizing YPPs and secreting these proteins and stored fats into the haemolymph, a time when water regulation may not be as vital. AaAQP1 and 2 immunoreactive staining recovers as the fat body reverts back to nutrient storage and lipid synthesis.

### AaAQP localization in the ovaries

The ovaries are the site for oocyte production and maturation. In *Ae. aegypti*, follicles in the ovaries containing oocytes are kept in a previtellogenic state until a blood meal is imbibed (38). Upon blood feeding the ovaries take up proteins and fats from the haemolymph, originating mainly from the fat body, to produce mature oocytes (37, 39). In general, oocyte maturation is also accompanied by water uptake in follicles and the AaAQP2 ortholog of *Bombyx mori* was implicated in this process (40). In particular, (40) showed that the highest expression of the AaAQP2 ortholog in *Bombyx mori* was during vitellogenesis, aiding in water transport and hydration of oocytes. Furthermore, the localization of an AaAQP1 ortholog in *B. mori* oocytes suggests that this AQP may function to prevent dehydration, as its highest expression is found during late-stage oogenesis known as choriogenesis, when the vitelline envelope is developed to protect the egg from water loss, which occurs ~2 days pre-eclosion (40). In the current study, AaAQP1 (DRIP ortholog) and AaAQP2 (PRIP ortholog) immunoreactive staining was identified in the ovaries of PBM mosquitoes (**Figure 1**), which is consistent with earlier reported transcript expression of these AQPs in the ovaries (13). Additionally, it was found that AgAQP1A, the ovarian-specific AaAQP2 ortholog in *Anopheles gambiae*, was found to be abundant in non-blood fed female ovaries, with significant increases in abundance 24–48hr post blood meal (41). Earlier, (34) examined AgAQP1 abundance *in vivo* and found that expression was significantly increased 48hr post blood feeding. Together, this suggests that both AaAQP1 and AaAQP2 may participate in water uptake during oocyte maturation or prevention of dehydration of oocytes in *Ae. aegypti*. Further studies to pinpoint the exact localization of these AQPs in the ovaries of *Ae. aegypti* is needed. Additionally, the current study found an entomoglyceroporin ortholog to the *D. melanogaster* Eglp1, known as AaAQP4, was immunolocalized in the ovaries after blood feeding (**Figure 2**). Relatively low levels of AaAQP4 transcript has been shown to be present in adult female *Ae. aegypti* ovaries, pre- and post-blood meal (16). A role for AaAQP4 in the ovaries is still unclear and requires further investigation.

### AaAQP localization in the midgut

The posterior midgut of the female mosquito stores and digests the blood meal and is the gut region where fluid and nutrients are absorbed into the haemolymph (42, 43). Immunoreactivity for AaAQP2 was found on the haemolymph-facing basolateral surface of the posterior midgut in 0.5hr PBM mosquitoes (**Figure 1E, E'**). Since the large volume load imbibed with a blood meal is absorbed into the haemolymph and subsequently secreted by the Malpighian tubules, basolaterally localized AaAQP2 may participate in transporting water across the midgut epithelium into the haemolymph during this time. Similarly, it was found that the PRIP ortholog, BcAQP2 in the insect *Bactericera cockerelli* was found to be highly expressed in the gut of the animal, likely responsible for transport of water post-food digestion (44). It has also been recently found that in *Ae. aegypti*, *aqp2* mRNA is significantly downregulated 3hr PBM (45). In addition, some immunoreactivity of AaAQP1 was found on the lumen-facing apical surface of the posterior midgut in 0.5hr PBM mosquitoes. It is possible that AaAQP1, a water-specific aquaporin, is important in the midgut epithelial tissue post blood feeding, to aid in removal of water from the midgut. Furthermore, various DRIP orthologs have been identified in the midgut of insects such as in larval midge *Belgica antarctica* (46), the rice striped stem borer, *Chilo suppressalis*, with confirmation of expression in whole adult female animals (47), 5^th^-instar *Rhodnius prolixus* posterior midgut samples (48), and in the columnar midgut cells of the silkworm *Bombyx mori* (49).

### AaAQP localization in the hindgut and rectal pads

The hindgut of adult female *Ae. aegypti* contains an elongated anterior segment, known as the ileum, and a more posterior region called the rectum, which contains six luminal rectal pads. Both segments have been implicated in ion and water absorption through the localization of major ion-transporting pumps such as Na^+^/K^+^-ATPase and V-type H^+^-ATPase (50). After a blood meal, alterations to the basolateral membrane of rectal pads suggest an increase in absorptive activity (51). Not surprisingly, transcript expression of AaAQP1 and AaAQP2 was detected in the ileum and rectum of female mosquitoes (13) and we detected immunoreactivity of both in the hindgut (**Figure 1**). Specifically, AaAQP1 was detected on the apical membrane of the ileum and in the apical membrane of the rectal pads (**Figure 1**) where it could play a role in absorbing water from the gut contents prior to excretion. In particular, the strong immunoreactive staining on the apical membrane of rectal pads 24hr PBM fits with the implied increased absorptive activities during this time (23, 52). AaAQP2 was detected in the ileum at 0.5hr PBM but, we could not verify if AaAQP2 was in the rectal pads because we did not have sections of rectal pads for NBF or 0.5hr PBM. Notably, AaAQP2 immunoreactivity was not observed in the rectal pads at 24hr PBM (**Figure 1F**), which is consistent with the very lower transcript abundance in the rectum reported earlier (13). Taken together, these findings indicate that AaAQP2 may also aid in water absorption shortly after the mosquito takes a blood meal.

### AaAQP localization in the Malpighian tubules

The Malpighian tubules (MTs) in insects are responsible for the production of ion-rich primary urine through the secretion of ions and water from the haemolymph. Using immunohistochemistry, we localized AaAQP1 (**Figures 1A–C**), 2 (**Figures 1D–F**), 4 (**Figures 2A–C**), and 5 (**Figures 2D–F**) in the MTs of adult female *Ae. aegypti* while AaAQP6 immunoreactivity was not detected in this organ (**Figures 1G–I**). We detected AaAQP2 immunoreactivity at the apical membrane of the MTs which manifested as discrete aggregated staining in the NBF group and more evenly distributed staining around the luminal circumference of the MT in post-blood fed mosquitoes (**Figures 1E, F**). There is a possibility that the aggregated staining is indicative of AaAQP2 expression in the small stellate cells of the MTs in NBF mosquitoes, although the evenly distributed staining observed after blood feeding is more akin to expression in the principal cells. AgAQP1, the AaAQP2 ortholog in *Anopheles gambiae*, was localized to the proximal principal cells as well as the distal stellate cells, in the MTs of NBF mosquitoes (34, 41). In *Drosophila melanogaster* MTs the expression of the ortholog of AaAQP2 (PRIP) is enriched in the stellate cells but is also expressed by principal cells (53, 54). The transition from aggregated staining to an even distribution of AaAQP2 at the apical membrane of the MTs after blood feeding suggests that expression of AaAQP2 in the MTs may become associated with the stellate cells as well as the principal cells, to deal with an increased demand for water transport through the tubule epithelium. This may be necessary because principal cells are the more abundant cell type in the MTs of *Ae. aegypti* making up the majority of the tubule, whereas the relatively small volume that stellate cells make up may not be able to handle the large volume of fluid secretion that occurs after blood feeding (55).

Entomoglyceroporin immunoreactivity was detected in the principal cells of the Malpighian tubules. AaAQP4 immunoreactivity was localized to the apical side while AaAQP5 immunoreactivity appeared at the basolateral membrane (**Figure 2**). Two entomoglyceroporin orthologs are also expressed in the principal cells of *Drosophila* MTs (53). In *Aedes albopictus* AQP4 transcript abundance is decreased with blood feeding in the Malpighian tubules while there is a short-lived increase in AQP5 transcript abundance (56). Conflicting observations have been reported in *Ae. aegypti* with both a short-lived increase in AaAQP4 transcript or a more sustained increase (13, 16). On the other hand, a sustained increase in AaAQP5 transcript was reported and later confirmed (13, 16). It appears that the increase in AaAQP4 mRNA may result in a greater protein abundance since we detected an increased intensity of AaAQP4 immunoreactive staining at 24hr PBM. We did not detect any changes in the intensity of AaAQP5 immunoreactive staining after blood feeding, which might suggest that the brief increase in *aqp5* mRNA seen by Esquivel et al. in (56), is required to maintain the baseline AaAQP5 protein levels. AaAQP5 was shown to be an efficient water transporter in a heterologous system and its knockdown increases adult mosquito survival under desiccation stress and reduces fluid secretion by MTs in larvae (13, 19).

Previous studies using an antibody generated against *Cicadella viridis* AQPcic reported that AQP1 expression is confined to the tracheolar cells of *Ae. aegypti* female MTs (20, 57); however, the antigen region which this antibody was generated against shares relatively low identity (4/15 residues, ~27%) with the *Ae. aegypti* AaAQP1. However, in the present study, using a custom AaAQP1 affinity-purified antibody, immunoreactivity was detected at the apical membrane of the MTs, showing aggregated staining under NBF conditions followed by distinct continuous staining along the circumference of the apical membrane of the MTs in PBM female mosquitoes (**Figures 1A–C**). AaAQP1 antibody specificity was confirmed by *de novo* confirmation of the full *aqp1* coding sequence with rapid amplification of cDNA ends (RACE), followed by the heterologous expression of AaAQP1 in HEK293T cells (see **Supplementary Results**). Through western blotting using our custom AaAQP1 antibody, expression of AaAQP1 in HEK293T cells revealed a single ~25kDa band, representing the putative AaAQP1 monomer (**Supplementary Figure S2**). Using FISH, the *aqp1* transcript was localized to the principal cells in NBF female MTs. Furthermore, subcellular localization of AaAQP1 in NBF female MTs using immunogold labeling found AaAQP1 dispersed within the principal cells, specifically to the extensive brush boarder which is thinner in stellate cells (**Figure 5**). In both *Ae. aegypti* and *Anopheles gambiae*, transcript abundance of AQP1 is significantly higher in blood fed versus sugar fed mosquito MTs suggesting that AaAQP1 is important in voiding the excess water load imbibed with a blood meal (13, 58), which is supported by reduced diuresis in AaAQP1 knockdown mosquitoes (16). Our findings show that staining of AaAQP1 in the MTs appears uniformly at the apical membrane after a blood meal but no changes in immunoreactive staining intensity were observed. TEM with immunogold labeling confirmed that AaAQP1 is predominantly localized to the principal cells of the MTs, although some presence was detected in the smaller and less abundant stellate cells. Through western blotting, we confirmed that the protein abundance of AaAQP1 in MTs is unchanged after blood feeding in comparison to non-blood fed mosquitoes (**Figure 6**). This raises the possibility that activity of AaAQP1 is regulated by translocation to the membrane and/or through post translational modifications rather than expression. A recent study by Kandel and colleagues examined AQP regulation in *Ae. aegypti* by phosphorylation and concluded that it is more likely that AQPs are regulated by membrane translocation during the post-blood feeding period in adult female mosquitoes (23).

In conclusion, this study established a comprehensive localization of AaAQP1, 2, 4, 5, and 6 in adult female *Ae. aegypti*, during NBF, 0.5hr PBM, and 24hr PBM conditions. In particular, AaAQP1 (DRIP ortholog) was localized to the MTs, fat body, ovaries, and hindgut pre- and post-blood feeding. It was also found that AaAQP1 is specifically localized to the principal cells of the MTs. Furthermore, AaAQP2 (PRIP ortholog) was localized to the MTs, fat body, ovaries, midgut, and hindgut. The entomoglyceroporin AaAQP4 (Eglp1 ortholog) was found primarily in the MTs, with immunoreactivity also observed in the fat body, ovaries, and hindgut. The entomoglyceroporin AaAQP5 (Eglp2 ortholog) was found primarily in the fat body, with minimal immunoreactivity also found in the MTs and ovaries. AaAQP6 immunoreactivity was found to be absent throughout the abdominal tissue sections, which aligns with earlier studies that demonstrated enrichment of this aquaporin within the foregut of the alimentary canal (16) that was not investigated herein. Further studies on the role of each AaAQP in the various organs of female *Ae. aegypti* will provide a better understanding of the mechanisms by which water and other solutes are transported within mosquitoes.

## Data availability statement

The original contributions presented in the study are included in the article/**Supplementary Material**, further inquiries can be directed to the corresponding authors/.

## Ethics statement

Ethical approval was not required for the studies on humans in accordance with the local legislation and institutional requirements because only commercially available established cell lines were used. Ethical approval was not required for the studies on animals in accordance with the local legislation and institutional requirements because only commercially available established cell lines were used.

## Author contributions

BP: Conceptualization, Data curation, Formal analysis, Investigation, Methodology, Project administration, Validation, Writing – original draft, Writing – review & editing. JP-P: Conceptualization, Funding acquisition, Resources, Supervision, Writing – review & editing. AD: Conceptualization, Funding acquisition, Resources, Supervision, Writing – review & editing.
