# Supplementary material for: Protein localization of aquaporins in the adult female disease vector mosquito, Aedes aegypti
Source: Front Insect Sci. 2024 Apr 18;4:1365651. doi: 10.3389/finsc.2024.1365651 (PMC11064791; doi:10.3389/finsc.2024.1365651)
Supplement: Supplementary file 1 [file DataSheet_1.pdf]

Supplementary Material

Supplementary Materials and Methods

AaAQP1 Sequence Confirmation and Heterologous Expression in HEK293T Cells

The current *aqp1* (AAEL024675) gene model in the improved reference genome of *Aedes aegypti* (Matthews et al., 2018) contains two annotated transcripts (AAEL024675-RA and AAEL024675-RB) that do not match the cloned sequence previously reported (Pietrantonio et al., 2000), leading to dissimilar deduced proteins. Importantly, these predicted transcripts yield a deduced protein that excludes ~90 amino acids on the N-terminal half of the protein and possessing a different C-terminus, which has implications for the custom antibody we designed that used a specific antigen on the C-terminus of the originally reported sequence (Pietrantonio et al., 2000). In order to independently determine and confirm the 3’ end of the *aqp1* gene in *A. aegypti*, specific primers were designed (Table S1). Malpighian tubule RNA was isolated from 4-day old adult females and cDNA was synthesized. Utilizing various reverse primer combinations (Table S1), a partial *aqp1* product containing the full open reading frame (ORF) and 273bp of the 3’ untranslated region, UTR) was successfully amplified by PCR using Q5® High-Fidelity DNA Polymerase and base accuracy was confirmed by Sanger sequencing revealing a partial *aqp1* matching that previously reported (Pietrantonio et al., 2000).

To improve the gene model and determine the transcripts derived from the *aqp1* gene, the FirstChoice® RLM-RACE kit (Thermo Fisher Scientific, Mississauga, ON) was utilized to amplify the complete 5’-UTR. Malpighian tubules were dissected from 4-day old adult female *A. aegypti* (n = 100) and total RNA was purified using the Monarch Total RNA Miniprep Kit (New England Biolabs, Whitby, ON). The RACE kit protocol was optimized for a small-scale reaction requiring 1µg of total RNA. For each nested PCR, both positive and negative control reactions were included involving two gene-specific primers with and without the template, respectively. Following successful amplification of *aqp1* with the 5’ inner RACE primer (provided in the kit) and two nested gene-specific reverse primers (Table S1), PCR products were purified using the Monarch DNA Gel Extraction kit (New England Biolabs, Whitby, ON) and ligated to pGEM T-Easy cloning vector. An aliquot of this ligation reaction was used to transform DH5α-strain *Escherichia coli* and grown on ampicillin-selective LB

plates (with X-GAL). Recombinant colonies were screened by colony PCR using small volume reactions (10µl) containing standard Taq polymerase and two gene-specific primers, specifically *aqp1* 5' RACE nested forward and *aqp1* 5' RACE reverse 2 (or *aqp1* 5' RACE reverse 3) (Table S1). The individual toothpicks used to screen isolated colonies on the plates were placed into microcentrifuge tubes containing 250µl of ampicillin selective LB media, and bacteria were grown overnight at 37°C, with constant shaking. The following day, an aliquot of each overnight culture was diluted 10-fold with sterile water and samples were re-screened with the 5' inner RACE primer (provided by kit) and 5' RACE reverse 2 (or *aqp1* 5' RACE reverse 3) primers. Several colonies that yielded the largest PCR product sizes (representing the most complete 5'UTR) were selected and larger scale bacterial cultures were grown overnight, shaking at 37°C. Plasmid DNA samples were then purified from the bacterial overnight cultures using the Monarch Plasmid Mini Prep kit (New England Biolabs, Whitby, ON, Canada) and their base characteristics were confirmed by Sanger sequencing (The Centre for Applied Genomics, Sick Kids, Toronto, ON, Canada).

Upon confirmation of the full *aqp1* sequence using RACE, the ORF was amplified with Q5® High-Fidelity DNA Polymerase. Reamplification was completed with a forward primer containing a start codon preceded by a Kozak consensus translation initiation sequence and *Hind*III restriction site and reverse primer with a stop codon and *Xba*I restriction site for directional cloning (Table S1). The PCR products were then purified with a Monarch PCR Clean Up Kit (New England Biolabs, Whitby, ON, Canada) following the kit protocols, and 1µg of each sample was digested (37°C for 40min) with the *Hind*III and *Xba*I restriction enzymes, using the Anza™ Red Buffer (Thermo Fisher Scientific, Burlington, ON, Canada). The digested products were then run on an agarose gel and purified by gel extraction using the Monarch Gel Extraction kit (New England Biolabs, Whitby, ON, Canada), following the kit protocols. The *aqp1* ORF product was then ligated into a mammalian expression vector (pcDNA3.1^{+}), using a 3:1 molar ratio of the *aqp1* insert to the plasmid, with the Anza™ T4 Ligase Master Mix (Thermo Fisher Scientific, Burlington, ON, Canada). The ligated products were transformed into NEB® 5-alpha Competent *E. coli* (New England Biolabs, Whitby, ON, Canada) and grown at 37°C overnight on ampicillin-selective LB plates. The following day, colonies containing the correct amplicon size were grown overnight in liquid culture before plasmid DNA samples were purified using the Monarch Plasmid Mini Prep kit (New England Biolabs, Whitby, ON, Canada). Prior to using these constructs in heterologous expression, base accuracy was confirmed with Sanger sequencing (The Centre for Applied Genomics, Sick Kids, Toronto, ON, Canada).

Human embryonic kidney (HEK293T) cells were grown in complete growth media (Dulbecco's modified eagles' medium: nutrient F12 (DMEM) media, 10% heat inactivated fetal bovine serum, and 1x antimycotic-antibiotic) within sterile T25 flasks, which were kept in an incubator at 37°C, 5% CO_2. Cells were monitored for growth and once they reached 100% confluency, the cells were re-seeded into a sterile 6-well plate (Thermo Fisher Scientific, Burlington, Ontario Canada) and left to grow overnight. The following morning, the cells were re-seeded at 90% confluency in a sterile 6-well plate and left to grow for 6hrs at 37°C. After the growth period, the cells were transfected with the mammalian expression *aqp1* construct previously made, in a 6-well plate. For transfection, Lipofectamine 3000 transfection reagent (Life Technologies, Carlsbad, CA, Unites States) was used in a 3:1 (µl:µg) ratio with DNA construct.

Preparation of Protein from Cell Samples

At 48hr after transfection, the complete media from each well of the 6-well plate was removed and placed in individual 15mL centrifuge tubes (Thermo Fisher Scientific, Burlington, ON, Canada) to collect any non-adherent cells floating in the culture media. Cells were detached from the plate surface using 2mL per well of sterile PBS/EDTA mixture (PBS + 5mM EDTA). After approximately 10min, the mixture containing the dissociated cells was then transferred to the centrifuge tubes, and samples were centrifuged at 400xg for 7 min at room temperature. The supernatant was discarded, and the cell pellets were re-suspended in 2mL per well of sterile PBS. Cell samples were centrifuged again at 400xg for 7 min at room temperature. The supernatant was discarded and then cells were re-suspended in 200µL of RIPA buffer with 1:100 protease inhibitor cocktail. Immediately after resuspension, cells in RIPA buffer were transferred to RNAse and DNAse-free microcentrifuge tubes and placed on ice. Cell samples were then sonicated at high frequency for ~15 sec, on ice. The samples were then loaded into 12% SDS-PAGE gels and the previously described western blot protocol (see Materials and Methods section in article) for AaAQP1 abundance was followed.

Supplementary Results

aqp1 Sequence Confirmation in Ae. aegypti and Heterologous Expression of aqp1 in Mammalian Cells

Utilizing a variety of molecular techniques, we confirmed that the sequence of *aqp1* in *Ae. aegypti* mosquitoes (Genbank accession#: PP003259) matches the previously described sequence

(Pietrantonio et al., 2000) and conclude that the current gene model and two predicted transcripts are inaccurate (https://vectorbase.org/vectorbase/app/record/gene/AAEL024675) in light of the empirically determined *A. aegypti aqp1* sequence in two separate studies. In addition, we utilized heterologous expression to confirm that our custom specific AaAQP1 antibody detects the ectopically expressed AaAQP1 protein while no such band is observed in HEK293T that do not express AaAQP1 (Figure S2). Using western blotting, we confirmed that our AaAQP1 antibody successfully bound to HEK293T-expressed protein with a band at the correct molecular weight of ~25kDa (Figure S2).

Supplementary Figures

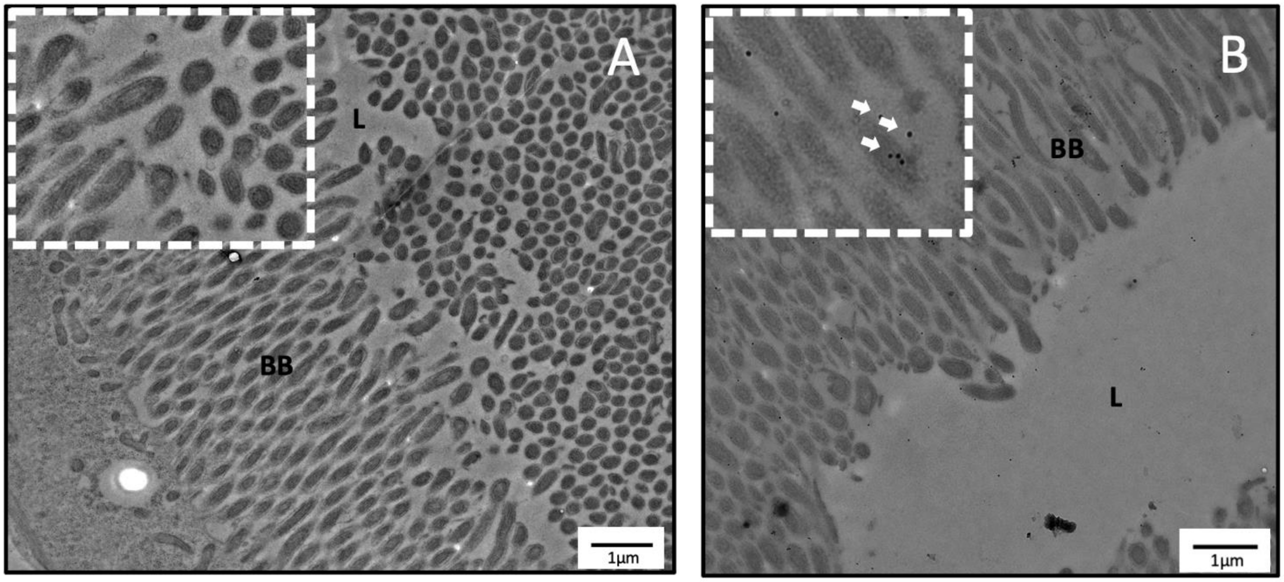

Figure S1. Control for TEM with Immunogold Localization of AaAQP1 in Female MT Principal Cells. **A.** showing control grid of MT principal cell tissue section, where a colloidal gold AffiniPure goat anti-rabbit secondary antibody conjugated with 18nm gold particles (Jackson Immunoresearch) was applied to samples and our specific AaAQP1 antibody was omitted. An absence of gold particles is seen, which can be better visualized in the higher magnification inset image enclosed in the white dashed lines. **B.** MT principal cell tissue section processed with our specific AaAQP1 antibody as well as the secondary antibody conjugated with 18nm gold particles, showing AaAQP1 localization scattered throughout the brush border. Goal particles can be visualized in the higher magnification inset image enclosed in the dashed white lines and indicated with individual white arrows.

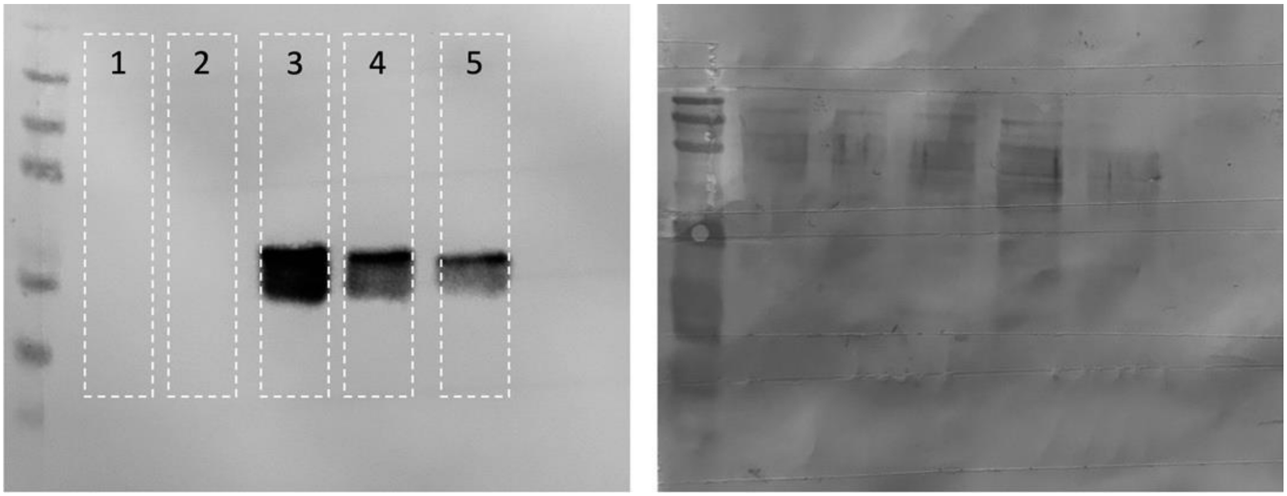

Figure S2. Verification of AaAQP1 Antibody Specificity with HEK293T cells expressing AaAQP1. Western blot analysis of AaAQP1 protein expressed and isolated from HEK293T cells (left), with associated Coomassie stained membrane for total protein (right). Lane 1 represents protein isolated from untransfected cells, with no band detected. Lane 2 represents protein isolated from HEK293T cells transfected with pcDNA3.1+ plasmid containing mCherry, with no band produced. Lanes 3-5 are separate independent transfections of HEK293T cells with the pcDNA3.1+ AaAQP1 construct, which was confirmed a single band at ~25kDa detected by the custom antibody for *Ae. aegypti* AQP1 (n=3).

Table S1. Oligonucleotides used for *Ae. aegypti aqp1* gene amplification including rapid amplification of cDNA ends (RACE), fluorescent *in situ* hybridization probe synthesis, and plasmid preparation for heterologous expression in HEK293T cells.

**Forward primer name & sequence:**	**Reverse primer name & sequence:**	**Function:**
aqp1 forward 1: GCTGTTTTACAAGGTCCCAT<br><br>*aqp1* forward 2: CAGCAGGAGAGACGAAAAGC	*aqp1* 3'end reverse: CCCGATGTATGATAGGCTCAAG	3' End Sequence Confirmation
aqp1 5' RACE nested forward: CGAGAACCGCAACATATGGC	*aqp1* 5' RACE reverse 1: CGTCGGTTAGTCACTTGTCC<br><br>*aqp1* 5' RACE reverse 2: AAGGGGAAAAATCAAGCACA<br><br>*aqp1* 5' RACE reverse 3: TTTTGGTTAGTGGGGGATGC	5' FirstChoice® RLM-RACE
aqp1 FISH forward: CGAGAACCGCCAACATATGGC	*aqp1* FISH reverse: TTACCCATAACGACGGCTGG	Fluorescent *In Situ* Hybridization probe synthesis
aqp1 start+**Kozak**: **GCCACC**ATGACTGAAAGCGCAGGC<br><br>*aqp1* start+**Kozak** +HindIII: AAGCTT**GCCACC**ATGACTGAAAGCGCAG	*aqp1* Stop+XbaI: TCTAGATTAAAAATCGTAAGATTCC	Plasmid construct with *aqp1* for heterologous expression in HEK293T cells

References

Drake, L.L., Boudko, D.Y., Marinotti, O., Carpenter, V.K., Dawe, A.L., Hansen, I.A. (2010). The aquaporin gene family of the yellow fever mosquito, *Aedes aegypti*. *PLOS One*. https://doi.org/10.1371/journal.pone.0015578

Matthews, B.J., Dudchenko, O., Kingan, S.B. et al. (2018). Improved reference genome of *Aedes aegypti* informs arbovirus vector control. *Nature* 563, 501–507. https://doi.org/10.1038/s41586-018-0692-z

Pietrantonio, P.V., Jagge, C., Keeley, L.L., Ross, L.S. (2000). Cloning of an aquaporin-like cDNA and in situ hybridization in adults of the mosquito *Aedes aegypti* (Diptera: Culicidae). *Insect Mol Biol*. (4):407-418. https://doi.org/10.1046/j.1365-2583.2000.00201.x
